# Genomic regions associated with bovine respiratory disease in pacific northwest Holstein cattle

**DOI:** 10.3389/fvets.2025.1637087

**Published:** 2025-07-31

**Authors:** Allison L. Herrick, Jennifer N. Kiser, Stephen N. White, Holly L. Neibergs

**Affiliations:** ^1^Department of Animal Sciences, Washington State University, Pullman, WA, United States; ^2^Washington Animal Disease Diagnostics Laboratory, Washington State University, Pullman, WA, United States; ^3^Department of Veterinary Microbiology and Pathology, Washington State University, Pullman, WA, United States; ^4^United States Department of Agriculture, Agricultural Research Service, Athens, GA, United States

**Keywords:** bovine respiratory disease, genome wide association analysis, gene set enrichment analysis, dairy cattle, shared genes

## Abstract

**Introduction:**

Bovine respiratory disease (BRD) is the leading natural cause of death in cattle. It is a multifactorial disease comprised of bacterial and viral pathogens. To aid in the reduction of BRD morbidity and mortality and the selection of cattle with reduced susceptibility, the objectives of this study were to identify loci, gene sets, positional candidate and leading-edge genes associated with or enriched for BRD in pre-weaned and post-weaned Holstein calves.

**Methods:**

From a single dairy, 518 pre-weaned (0–60 days old) and 2,001 post-weaned (61–421 days old) Holstein heifers were treated for BRD and served as cases. All 3,655 pre-weaned healthy control calves remained in the herd for a minimum of 60 days, and 3,210 healthy post-weaned control calves remained in the herd for a minimum of 421 days. Loci associated (uncorrected *p* < 5 **×** 10^−7^) with BRD were identified using EMMAX with additive, dominant and recessive inheritance models. Positional candidate genes were identified within a haplotype of an associated SNP. A GSEA-SNP was performed to identify gene sets (NES ≥ 3) and leading-edge genes enriched for BRD.

**Results:**

There were four additive, six dominant, and three recessive loci associated (*p* < 5 **×** 10^−7^) with BRD in pre-weaned calves and 22 additive, 17 dominant, and 13 recessive loci associated with BRD in post-weaned calves. SNPs associated with pre-weaned BRD were within 26 positional candidate genes and 56 positional candidate genes in post-weaned calves. Heritability was estimated as 0.16 ± 0.02 for both groups. One gene set with 86 leading-edge genes was enriched (NES = 3.13) for the pre-weaned calves while 7 gene sets with 162 unique leading-edge genes were enriched (NES ≥ 3) in the post-weaned calves. The positional candidate genes, *EBF1* and *SPAG16*, and the leading-edge gene *COL4A3BP* were shared between the pre-and post-weaned calves, which have functions related to inflammation and immune cell development. The identification of loci, gene sets, positional candidate and leading-edge genes associated and enriched for BRD in different ages of dairy calves provides a better understanding of the disease process and facilitates selection for animals more resistant to this complex disease.

## 1 Introduction

Bovine respiratory disease (BRD) is one of the most common and expensive infectious diseases impacting cattle throughout the United States, with economic losses estimated at over three-billion dollars annually, and among dairy calves, an estimated cost per case of approximately $282 (1–3). In addition to the initial cost of treatment and labor needed for treating cattle, cattle experiencing BRD are more likely to have reduced performance throughout the rest of their lives, extending the cost of the disease (4, 5). Bovine respiratory disease can be attributed to commensal and pathogenic bacteria (*Mannheimia haemolytica, Pasteurella multocida, Histophilus somni, Mycoplasma bovis*, and *Trueperella pyogenes*) and viruses (bovine respiratory syncytial virus, bovine herpesvirus 1, and bovine viral diarrhea virus) (6–8). Pathogen and BRD prevalence will vary by the environment, management, weather, hygiene and stress that the cattle are exposed to USDA (9). Specifically looking within the dairy industry, identifying ways to reduce the prevalence of BRD is essential to establish healthier and more profitable dairy herds. Moreover, consumers are increasing their desire for dairy products that have come from cattle with limited antimicrobial use, and BRD has been associated with anti-microbial treatments in 11.4% of pre-weaned heifers and in 4.7% of post-weaned heifers (10).

Management practices that have been implemented to reduce BRD prevalence have included vaccination programs, better colostrum management, infrastructure of facilities to improve ventilation and hygiene, and animal handling practices to reduce stress (11). Although these practices have benefited the dairy calf, the incidence of BRD has not declined. As BRD susceptibility has a genetic component, the identification of loci associated with BRD susceptibility has been an active area of research in beef and dairy cattle to select those with enhanced resistance to the disease (12–14). As the pathogens, management and environmental factors associated with BRD prevalence will differ across dairies, validation of loci associated with BRD is critical and can provide additional information for selection for enhanced BRD resistance. Therefore, the objective of this study was to identify loci and positional candidate genes associated with BRD, and gene sets and leading-edge genes enriched for BRD in pre-weaned and post-weaned Holstein calves.

## 2 Materials and methods

### 2.1 Study population

This study (#6743) was approved by the Institutional Animal Care and Use Committee of Washington State University. A single Idaho dairy provided Dairy Comp 305 (Valley Agricultural Software, Tulare, CA, United States) records and Zoetis CLARIFIDE^®^ Plus (Zoetis Precision Animal Health, Parsippany, NJ, United States) genotypes of 6,423 Holstein calves. Cattle within the study were born over an eight-year period from a single dairy that milks approximately 2,300 cows. Cattle in the study were from a single herd at a single location in Idaho. Cattle were housed in dry lots with shade and were fed a total mixed ration. All cattle had Dairy Comp 305 records of animal events, health records, and management notes that were used for the study.

The study consisted of a pre-weaned (birth to 60 days of age) calf group and a post-weaned (61–420 days of age) calf group, encompassing the two critical development periods between birth to approximate insemination (14 months). Cases were identified as calves that were treated for BRD or had a recorded respiratory event in the pre-weaned (*n* = 518) or post-weaned period (*n* = 2,001). Due to the use of retrospective records, no clinical diagnoses, assessments, or bacteriology/virology were assessed within this study. Diagnosis was solely based upon BRD events, and animals were not removed based upon additional health events, including diarrhea. Control calves were required to remain in the herd for the entirety of the period in which the cases were identified. For the 3,655 healthy, pre-weaned controls, all calves remained and were observed for disease until at least 60 days of age. For the 3,210 healthy post-weaned controls, all calves remained in the herd and were observed for disease for a minimum of 421 days.

### 2.2 Genotyping

Genotypes from Zoetis CLARIFIDE^®^ Plus (Zoetis Precision Animal Health, Parsippany, NJ, United States) tests were imputed to approximately 620,000 SNPs using a single-step imputation process through Beagle v.4.0 (15) and the ARS-UCD 1.2 assembly (accessed on 8 January 2024).^1^ The imputation used a reference population that consisted of roughly 4,800 Holsteins from Washington, Idaho, California, and New Mexico and were genotyped using Illumina BovineHD BeadChip (San Diego, CA, United States). Study animals shared between 29,741 and 53,594 SNPs with the individuals from the reference population and the BovineHD BeadChip. To assess the accuracy of the imputation, previously genotyped animals were also included within the analysis and compared to their known genotypes, determining the final imputation accuracy to be calculated at > 95%. These genotypes were used for the genome-wide association analysis (GWAA) and the gene set enrichment analysis using SNP data (GSEA-SNP).

### 2.3 Quality control

Prior to GWAA, quality control was completed on the imputed SNPs and the animals genotyped for the pre-weaned and the post-weaned calves. Prior to quality control filtering, the pre-weaned population consisted of 4,171 individuals and 619,410 SNPs and the post-weaned population consisted of 5,211 individuals and 619,410 SNPs. Imputed genotypes were removed if the call rate < 0.9 (*n* = 6,421 for pre-weaned, *n* = 6,415 for post-weaned), if they had a minor allele frequency < 0.01 (*n* = 99,048 for pre-weaned, and *n* = 98,630 for post-weaned), or if they failed Hardy–Weinberg equilibrium testing with *p* < 1 × 10^−160^ (*n* = 5,985 for pre-weaned, and *n* = 7,086 for post-weaned). No calves were removed from the pre-weaned or post-weaned group for more than 10% of genotypes failing. After quality control filtering, there were 507,956 SNPs in the pre-weaned heifer analyses and 507,279 SNPs in the post-weaned heifer analyses.

### 2.4 Genome-wide association analysis

To identify population stratification, a principal component analysis (PCA) was completed for the pre-weaned and post-weaned calves. Clustering by birth year was identified for pre-and post-weaned calves and was included as a covariate in the GWAA. A genomic inflation factor (λ_GC_) was calculated to identify the level of population stratification with the use of birth year as a covariate (16). The SNP and Variation Suite (SVS) software version 8.1 (Golden Helix, Bozeman, MT, United States) was used for the GWAA. An efficient mixed-model association eXpedited statistical approach was used to perform the GWAA with an identity-by-state relationship matrix. The general EMMAX statistical approach is defined as y=Χβ+Ζu+∈ , where y = a *n* x 1 vector of observed phenotypic values, Χ = an *n* x *f* matrix for fixed effects, β = f x 1 vector for the coefficients of fixed effects, Ζ = a matrix containing random effects, u = a vector of random effects with variants of allele substitutions in the population, and ∈ = residual effects (17). Additive, dominant and recessive inheritance models were performed for the GWAA. Associations were established using the Wellcome Trust threshold for uncorrected *p*-values, with *p* < 1 × 10^−5^ providing evidence for a moderate association and *p* < 5 × 10^−7^ providing evidence for a strong association (18). The proportion of variance explained for each SNP was calculated within SVS for each GWAA. As the proportion of variance explained is not independent for all SNPs, the sum of these effects will exceed 100%. For SNP associated with BRD that were near one another, a locus was characterized when D′ > 0.7 (19, 20). Heritability was estimated within SVS using an AI-REML analysis, which is calculated with a matrix of allele substitution marker effects, otherwise known as a genomic best linear unbiased prediction (GBLUP) (21, 22).

### 2.5 Positional candidate genes

The average haplotype size for the calves in this study was 30,235 bp when calculated using the method of Gabriel et al. (23). Positional candidate genes were identified within one haplotype (± 30 kb) from the associated SNP based on the ARS-UCD 1.2 bovine genome assembly.^2^

### 2.6 Gene set enrichment analysis–single nucleotide polymorphism

The imputed genotypes were used to conduct the association analysis to identify the SNPs that would serve as representatives for the genes in the gene sets in BioCarta (217 gene sets),^3^ Gene Ontology or GO (3,147 gene sets),^4^ Kyoto Encyclopedia of Gene and Genomes or KEGG (186 gene sets),^5^ Protein Analysis Through Evolutionary Relationships or PANTHER (165 gene sets),^6^ and Reactome or R (674 gene sets).^7^ The quality control filtered SNPs in the pre-weaned and post-weaned heifer analyses were analyzed and mapped to 21,039 protein-coding genes within the ARS-UCD 1.2 genome assembly. SNPs with the greatest evidence of an association with BRD were used as gene proxies for the GSEA-SNP. Only one SNP was used to represent each gene, noting that a SNP may be a proxy for multiple genes. Genes were ranked by their *p*-value for their association with BRD from the association analysis. The analysis was run within R v. 3.6.3, using the GenGen v. 1 package (24). An enrichment score for each gene set was computed from the running sum statistics with each gene set receiving a permuted p-value after 10,000 phenotype-based permutations in GenABEL v. 3.6.3 (25, 26). The maximum enrichment score was normalized by the number of genes in the gene set, and those gene sets enriched for BRD had a normalized enrichment score (NES) ≥ 3.0, using a methodology as described by Neupane et al. (27). Leading-edge genes were those that contributed to the peak enrichment score.

## 3 Results

### 3.1 Genome-wide association analysis

**Figure 1:**
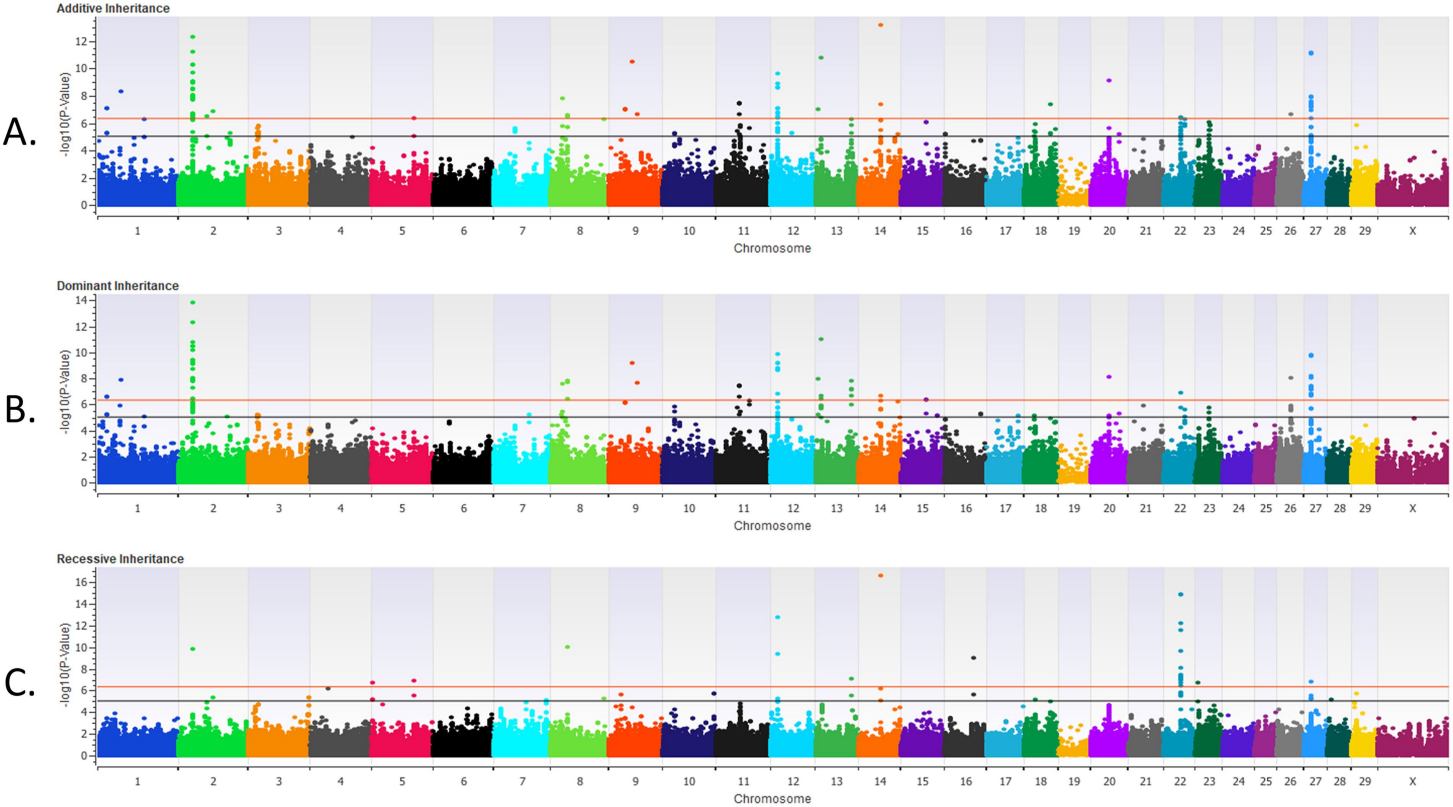
The results for the genome-wide association analysis for bovine respiratory disease are shown for the pre-weaned Holstein heifer calves for the additive inheritance model in **(A)**, the dominant inheritance model in **(B)** and the recessive inheritance model in **(C)**. Each Manhattan plot has the *Bos taurus* chromosomes on the x axis and the −log10 *p*-value on the y axis. The significance thresholds for an association with BRD are represented by the lower or black line (moderate association with an uncorrected *p* < 1 × 10^−5^) and the uppermost or red line (strong association with an uncorrected *p* < 5 × 10^−7^).

There were four loci strongly associated (*p* < 5 × 10^−7^) with BRD in the additive inheritance model, six loci strongly associated with BRD (*p* < 5 × 10^−7^) in the dominant inheritance model, and three loci strongly associated (*p* < 5 × 10^−7^) with BRD in the recessive model in the pre-weaned calves (Table 1 and Figure 1). Two loci, on BTA4 and on BTA7, were strongly associated (*p* < 5 × 10^−7^) with BRD in both the additive and dominant inheritance models. There were 16 additional loci moderately associated (*p* < 1 × 10^−5^) with BRD in the additive model, 11 loci moderately associated (*p* < 1 × 10^−5^) with BRD in the dominant model, and 10 loci moderately associated (*p* < 1 × 10^−5^) with BRD in the recessive model for the pre-weaned calves. The λ_GC_ values for the pre-weaned BRD population were 0.996 for the additive model, 0.994 for the dominant model, and 0.990 for the recessive model. There were 26 positional candidate genes where an associated SNP fell within an exon or intron of the gene, and an additional 39 positional candidate genes located within a haplotype of a SNP associated with BRD. The estimated heritability for BRD was 0.16 ± 0.02 for the pre-weaned calves.

**Table 1:** Genome wide association analysis results of loci strongly associated (*p* < 5 × 10^−7^) with BRD in pre-weaned Holstein heifer calves.

BTA^1^	# Associated SNPs^2^	Mb^3^	*p*-value^4^	Inheritance model^5^	Positional candidate genes^6^
4	3	80	4.86 × 10^−8^	Additive	***SUGCT***
5	8	46	2.37 × 10^−7^	Additive	*DYRK2, LOC112446699*
7	1	71	1.95 × 10^−9^	Additive	***EBF1***
9	3	55	4.46 × 10^−7^	Additive	*–*
1	3	71	2.25 × 10^−7^	Dominant	***NCBP2**, NCBP2-AS2, **PIGZ**, SENP5*
4	3	80	4.95 × 10^−8^	Dominant	***SUGCT***
7	1	71	1.55 × 10^−7^	Dominant	***EBF1***
9	4	55–64	4.67 × 10^−7^	Dominant	*–*
26	3	19	1.03 × 10^−7^	Dominant	***CRTAC1***
X	1	2	1.99 × 10^−7^	Dominant	*-*
13	6	74	2.73 × 10^−9^	Recessive	***ACOT8, CDH22,** CTSA, DNTTIP1, LOC104973895, **LOC112449338,** LOC112449416, NEURL2, PCIF1, **PLTP**, SNX21, SPATA25, TNNC2, UBE2C, WFDC10A, WFDC11, **WFDC13**, WFDC9, ZSWIM1, ZSWIM3*
14	2	27	3.36 × 10^−7^	Recessive	*–*
23	5	9	4.41 × 10^−7^	Recessive	***PPARD***

^1^*Bos taurus* chromosome (BTA) where the locus is associated with BRD in pre-weaned calves. ^2^Number of single nucleotide polymorphisms (SNPs) present within each associated locus. ^3^Megabase (Mb) position of each locus. ^4^The uncorrected p-value for the leading SNP for each locus. ^5^The inheritance model where each locus was associated with pre-weaned BRD. ^6^Positional candidate genes are genes that were identified within ± 30 kb (5′ and 3′) of associated SNPs. Bolded positional candidate genes have one of the associated SNPs located within it.

**Figure 2:**
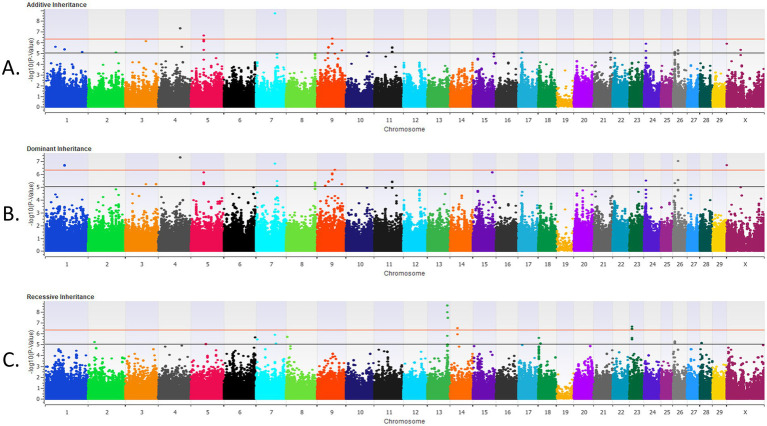
The results for the genome-wide association analysis for bovine respiratory disease are shown for the post-weaned Holstein heifer calves for the additive inheritance model in **(A)**, the dominant inheritance model in **(B)** and the recessive inheritance model in **(C)**. Each Manhattan plot has the *Bos taurus* chromosomes on the x axis and the −log10 p-value on the y axis. The significance thresholds for an association with BRD are represented by the lower or black line (moderate association with an uncorrected *p* < 1 × 10^−5^) and the top or red line (strong association with an uncorrected *p* < 5 × 10^−7^).

In the post-weaned calves, 21 loci were strongly associated (*p* < 5 × 10^−7^) with BRD with the additive inheritance model, 18 loci were strongly associated (*p* < 5 × 10^−7^) with BRD with the dominant inheritance model, and 11 loci were strongly associated (*p* < 5 × 10^−7^) with BRD in the recessive inheritance model (Table 2 and Figure 2). Eleven of the loci associated (*p* < 5 × 10^−7^) with BRD in the additive and dominant inheritance models were shared, six loci were associated (*p* < 5 × 10^−7^) in all inheritance models and a single locus associated (*p* < 5 × 10^−7^) with BRD on BTA13 was shared among all three inheritance models. In addition, there were 22 loci moderately associated (*p* < 1 × 10^−5^) with BRD in the additive model, 19 loci associated (*p* < 1 × 10^−5^) with BRD in the dominant inheritance model and 15 loci associated (*p* < 1 × 10^−5^) in the recessive model. The λ_GC_ values for the post-weaned BRD population were 0.986, 0.995, and 0.996 for the additive, dominant, and recessive models, respectively. There were 56 positional candidate genes where the associated SNP fell within an exon or intron of the gene, and an additional 88 positional candidate genes located within one haplotype of the SNP associated with BRD in the post-weaned calves. The estimated heritability was 0.16 ± 0.02 for BRD in the post-weaned calves.

**Table 2:** Genome wide association analysis results of loci strongly associated (*p* < 5 × 10^−7^) with BRD in post-weaned Holstein heifer calves.

BTA^1^	# Associated SNPs^2^	Mb^3^	p-value^4^	Inheritance model^5^	Positional candidate genes^6^
1	5	19	8.88 × 10^−8^	Additive	*CHODL*
1	1	46	5.16 × 10^−9^	Additive	***SENP7***
2	43	27	5.67 × 10^−13^	Additive	***CERS6**, LOC112443601, **NOSTRIN**, SPC25*
2	2	56	3.20 × 10^−7^	Additive	*TRNAC-GCA*
2	1	68	1.50 × 10^−7^	Additive	*LOC112443654*
5	2	83	4.57 × 10^−7^	Additive	***ITPR2***
8	2	24	1.82 × 10^−8^	Additive	***FOCAD**, MIR491*
8	8	33	3.15 × 10^−7^	Additive	*LOC112447904*
9	5	33	3.66 × 10^−11^	Additive	***DCBLD1**, LOC112448032*
9	1	57	2.51 × 10^−7^	Additive	*-*
11	7	47	3.91 × 10^−8^	Additive	***LOC100294952***
12	24	14	2.49 × 10^−10^	Additive	*LACC1, LOC112449035, **SERP2**, **SMIM2**, TSC22D1*
13	1	5	9.83 × 10^−8^	Additive	*-*
13	1	10	1.80 × 10^−11^	Additive	*-*
14	6	44	6.95 × 10^−14^	Additive	*LOC100295528, LOC112449520, LOC785035, **ZBTB10**, **ZNF704***
18	3	51	4.38 × 10^−8^	Additive	***CNFN**, LIPE, **MEGF8***
20	3	36	8.26 × 10^−10^	Additive	***EGFLAM**, LOC101905359*
22	12	33	4.66 × 10^−7^	Additive	*ARL6IP5, **EOGT**, **FAM19A4**, **TMF1**, **UBA3***
26	1	29	2.63 × 10^−7^	Additive	*-*
27	3	14	7.43 × 10^−8^	Additive	*DCTD, **LOC100848319***
27	2	14	4.77 × 10^−7^	Additive	*LOC112444630, LOC536739, **WWC2***
27	12	14	9.64 × 10^−12^	Additive	*CASP3, CDKN2AIP, **ENPP6,** ING2, LOC101903828, LOC101904332, LOC101907089, LOC104976048, **PRIMPOL, STOX2,** TRAPPC11*
1	5	19	2.63 × 10^−7^	Dominant	*CHODL*
1	2	45	1.50 × 10^−8^	Dominant	*ABI3BP, **SENP7***
2	42	27	1.52 × 10^−14^	Dominant	***CERS6**, LOC112443601, **NOSTRIN**, SPC25*
8	10	23–32	3.04 × 10^−8^	Dominant	*CDKN2A, CDKN2B, **FOCAD**, LOC101907577, LOC112447904, MIR491*
9	5	33	7.11 × 10^−10^	Dominant	***DCBLD1**, LOC112448032*
9	1	57	2.41 × 10^−8^	Dominant	*-*
11	7	47	3.79 × 10^−8^	Dominant	*LOC100294952, LOC784634, RPIA*
12	14	14	7.22 × 10^−10^	Dominant	*LOC112449035, **SERP2**, TSC22D1*
13	1	5	1.26 × 10^−8^	Dominant	*-*
13	8	10	9.96 × 10^−12^	Dominant	*-*
13	5	70	7.67 × 10^−8^	Dominant	***CHD6**, LOC112449381*
14	5	44	2.39 × 10^−7^	Dominant	*LOC112449520, LOC785035, **ZBTB10**, **ZNF704***
15	3	50	4.23 × 10^−7^	Dominant	*LOC112441490, LOC112441705, LOC618010, LOC782428, **LOC784976**, LOC785036, **LOC788946**, OR51A7, OR51E2, OR51T1*
20	2	36	8.23 × 10^−9^	Dominant	***EGFLAM***
22	1	33	1.39 × 10^−7^	Dominant	***FAM19A4***
26	7	29	9.12 × 10^−9^	Dominant	*-*
27	17	14	2.12 × 10^−10^	Dominant	*DCTD, **ENPP6**, **LOC100848319**, LOC101904332, LOC104976048, LOC112444630, LOC536739, **STOX2**, TRAPPC11, **WWC2***
0	1	0	1.58 × 10^−8^	Recessive	*-*
2	1	27	1.83 × 10^−10^	Recessive	***CERS6***
5	3	1	2.09 × 10^−7^	Recessive	*-*
5	2	83	1.51 × 10^−7^	Recessive	***ITPR2***
8	1	33	1.09 × 10^−10^	Recessive	*-*
12	1	14	2.26 × 10^−13^	Recessive	*-*
12	1	14	4.86 × 10^−10^	Recessive	***SMIM2***
13	2	70	8.22 × 10^−8^	Recessive	***CHD6***
14	3	44	2.82 × 10^−17^	Recessive	*LOC100295528, LOC101905394, **ZBTB10**, **ZNF704***
16	2	58	1.08 × 10^−9^	Recessive	*LOC112441790, **PAPPA2***
22	24	33	1.68 × 10^−15^	Recessive	*ARL6IP5, **EOGT**, **FAM19A4**, FRMD4B, LOC112443429, **TMF1**, **UBA3***
23	1	6	2.01 × 10^−7^	Recessive	*-*
27	1	14	1.57 × 10^−7^	Recessive	***TENM3***

^1^*Bos taurus* chromosome (BTA) where the locus is associated with BRD in post-weaned calves. ^2^Number of single nucleotide polymorphisms (SNPs) present within each associated locus. ^3^Megabase (Mb) position of each locus. ^4^The uncorrected *p*-value for the lead SNP at each locus. ^5^The inheritance model where each locus was associated with post-weaned BRD. ^6^Positional candidate genes are genes that were identified within ± 30 kb (5′ or 3′) of associated SNPs. Bolded positional candidate genes have one of the associated SNPs located within it.

### 3.2 GSEA-SNP results

The Reactome gene set, Phospholipid Metabolism, was enriched (NES = 3.128) for BRD in pre-weaned calves and contained 86 leading edge genes (Table 3). Seven gene sets were enriched (NES ≥ 3) for BRD in the post-weaned calves and consisted of 266 leading edge genes (Table 3). The leading edge gene, p21-activated kinase 1 (*PAK1*), was present in four of the gene sets enriched for BRD in the post-weaned calves, while FYN proto-oncogene (*FYN*), cyclin-dependent kinase inhibitor 2B (*CDKN2B*), cathepsin H (*CTSH*), casein kinase 2 alpha 1 (*CSNK2A1*), and integrin subunit beta 1 (*ITGB1*) were present in three gene sets in post-weaned calves. An additional 90 leading-edge genes were enriched in two gene sets in post-weaned calves. Only a single leading-edge gene, collagen type IV alpha-3-binding protein, also known as ceramide transfer protein or StAR-related lipid transfer protein (*COL4A3BP*), was shared as a leading-edge gene for BRD in the pre-and post-weaned calves.

**Table 3:** Gene set enrichment analysis—single nucleotide polymorphism results from pre-weaned and post-weaned Holstein heifer calves.

Enriched gene sets	Database	NES^1^	# of LEGs^2^	Leading edge genes^3^
Pre-weaned heifers				
Phospholipid metabolism	Reactome	3.13	86	*ACER1, ACER2, AGPAT1, AGPAT2, AGPAT3, ARF1, ARSB, ARSE, ARSH, ARSJ, ASAH2, CDS2, CEPT1, CERK, CERS3, CERS6, CHAT, CHKA, CHKB, CHPT1, COL4A3BP, CTSA, DGAT2, Fig 4, GBA3, GLB1, GM2A, GNPAT, GPAT2, GPD1L, HEXA, HEXB, INPP4B, INPP5J, LCLAT1, LPCAT1, LPCAT3, LPCAT4, MBOAT1, MBOAT2, MTM1, MTMR1, MTMR14, MTMR2, NEU1, NEU2, PCYT1A, PCYT1B, PI4K2A, PI4K2B, PI4KA, PIK3C2G, PIK3C3, PIK3CB, PIK3R1, PIK3R2, PIK3R3, PIK3R4, PITPNB, PLA2G12A, PLA2G16, PLA2G2A, PLA2G2E, PLA2G3, PLA2G5, PLBD1, PLD1, PLD4, PTEN, SACM1L, SGMS1, SGPL1, SGPP2, SLC44A1, SLC44A2, SLC44A4, SLC44A5, SMPD2, SMPD4, SPHK2, SPTLC2, STS, SYNJ2, TAZ, VAC14, VAPA*
Post-weaned heifers				
Axon guidance mediated by semaphorins	Panther	3.52	13	*ARHGEF1, CRMP1, DPYSL2, DPYSL5, DUSP5, **FYN**, NRP1, **PAK1**, PAK2, PLXNB1, RAC1, SEMA3A, SEMA4D,*
G1 phase	Reactome	3.44	9	*CDK6, CDKN2A, **CDKN2B**, E2F1, PPP2R1A, PPP2R1B, RB1, RBL1, RBL2*
Signal transduction by L1	Reactome	3.18	15	*AP2A1, **CSNK2A1**, CSNK2B, ITGA9, **ITGB1**, MAP2K1, NCAM1, NRP1, **PAK1**, RAC1, RPS6KA1, RPS6KA2, RPS6KA3, RPS6KA4, SH3GL2*
Cysteine type peptidase activity	Gene Ontology	3.35	12	*CAPN1, CASP3, CASP6, **CTSH**, CTSK, SENP7, TINAG, USP15, USP16, USP2, USP42, YOD1*
Platelet aggregation	Gene Ontology	3.18	2	*CLIC1, PRKG1*
Cell proliferation	Gene Ontology	3.16	120	*AIF1, ANG, ASCC3, ASPH, B4GALT1, BCL2, BCL2L2, BDNF, CASR, CCND1, CD3E, CD79A, CDCA7, CDH1, CDH3, **CDKN2B**, CEBPA, CEP120, CFDP1, CHRNA7, COL4A3BP, **CSNK2A1**, **CTSH**, CXADR, CXCL2, DNMT1, DPT, EDN1, EIF2S2, EMP2, EMX2, EPO, F2R, F2RL1, F3, FADD, FAP, FGF2, FGFBP1, FOXO1, FOXP1, **FYN**, GATA3, GHRL, GJA1, GPLD1, HOXA3, HOXA5, HP1BP3, IFNW1, IGF2, IGFBP2, IGFBP3, IGFBP5, IL10, IL15, IL3, IL34, IMPDH1, IMPDH2, ING4, INHBA, INTU, **ITGB1**, JUNB, KANK2, KCNH1, KITLG, LOXL2, LTF, MAPK1, MEF2C, MEN1, MSX2, MYOG, NCF1, NR4A1, **PAK1**, PAX6, PBLD, PDGFA, PHB, PIN1, PKD2, PLAC8, PPARG, PRDX3, PRDX4, PRNP, PSEN1, RAPGEF2, RBPJ, ROGDI, ROMO1, S1PR1, SBDS, SFN, SKAP2, SMAD1, SMARCB1, SMYD2, SRRT, SRSF6, STC1, STK4, STRAP, TAF8, TEK, THAP1, THBS4, TIPIN, TNF, UBE2L3, UHRF1, VDR, WDR6, WNT16, ZNHIT1, ZP3, ZP4*
Regulation of cell proliferation	Gene Ontology	3.15	95	*AIF1, ANG, ASPH, B4GALT1, BDNF, CASR, CCND1, CD3E, CDCA7, CDH1, CDH3, **CDKN2B**, CEBPA, CFDP1, CHRNA7, **CSNK2A1**, **CTSH**, CXADR, CXCL2, DNMT1, DPT, EDN1, EMP2, EPO, F2R, F3, FADD, FAP, FGF2, FGFBP1, FOXO1, FOXP1, **FYN**, GATA3, GHRL, GJA1, GPLD1, HOXA3, HOXA5, HP1BP3, IGF2, IGFBP2, IGFBP3, IGFBP5, IL10, IL15, IL3, IL34, ING4, INHBA, INTU, **ITGB1**, JUNB, KANK2, KCNH1, KITLG, LTF, MEF2C, MEN1, MSX2, MYOG, NR4A1, **PAK1**, PAX6, PBLD, PDGFA, PHB, PIN1, PKD2, PLAC8, PPARG, PRDX3, PRDX4, PRNP, RAPGEF2, RBPJ, ROGDI, ROMO1, S1PR1, SFN, SKAP2, SMAD1, SMARCB1, SMYD2, SRSF6, STK4, STRAP, THBS4, TIPIN, TNF, VDR, WDR6, ZNHIT1, ZP3, ZP4*

^1^Normalized enrichment score (NES). ^2^Total number of leading-edge genes (LEGs) within the gene set. ^3^Leading-edge gene symbols from each gene set. Bolded leading-edge genes are present in more than two gene sets.

## 4 Discussion

The identification of loci associated with BRD, and the positional candidate genes located at those loci, provide potential targets for genomic selection to reduce the incidence of BRD and provides insights into the etiology of the disease. The identification of gene sets and leading-edge genes enriched for BRD provides a broader view of how genes interact with one another to elicit a response to disease challenges. The use of different inheritance models and ages to identify these genomic regions involved in enhanced disease resistance or susceptibility further illustrates the complexity of the disease.

It is possible that other health conditions that occurred prior to diagnosis of BRD may have resulted in a more disease susceptible animal, but from the health records provided, it is not possible to clearly distinguish this possibility. The use of health records by the dairy to identify BRD does not exclude or necessarily provide information on other clinical diseases or pathological conditions that may be present in an animal with BRD. It is also important to note that inferences as to the cause and effect of corresponding conditions cannot be sorted out. However, this is also likely to represent how dairies will judge whether genomic selection for BRD is being reduced by comparing their records for BRD cases before and after selection, without correcting for other clinical diseases. Using this definition of a BRD case as being noted in the health records, only the most robust loci will be identified as associated with BRD as there is some possibility of confounding with increased susceptibility due to a concomitant disease process. The most common health issue described at this dairy for these animals was diarrhea and so the frequency of it coinciding with BRD was examined.

BRD and diarrhea occurred in the same animal in 14% of pre-weaned calves and 53% of post-weaned calves. When examining the ages of treatment for individuals who experienced both conditions, 95% of them experienced an event of diarrhea prior to contracting BRD, and the remaining 5% experienced a BRD event prior to an event of diarrhea. It is also unknown whether the diarrhea was due to BVDV, and so part of BRD (and not another distinct disease process) as the time/age of first symptoms was not documented.

### 4.1 Loci associated with bovine respiratory disease

This study identified a total of 156 loci across all models for both pre-and post-weaned BRD populations. Previous studies have estimated the heritability of cattle respiratory disease traits to be between 0.04 and 0.28 (14, 28, 29). The estimated heritability within this population was 0.16 for both the pre-and post-weaned calves, which fell within the range of heritabilities previously reported. The estimated heritability of BRD for pre-and post-weaned calves provides encouragement that selection for BRD will be beneficial in reducing disease morbidity and mortality.

### 4.2 Positional candidate genes

#### 4.2.1 Pre-weaned Holstein calves

There were 65 positional candidate genes found within loci associated with the pre-weaned BRD population (Table 1). To focus the discussion, only those positional candidate genes associated with loci in strong association (*p* < 5 **×** 10^−7^) with BRD that contain the associated SNP within an intron or exon, will be discussed further. Complete results for all moderately and strongly associated loci and positional candidate genes are included in Supplementary Table 1.

The greatest evidence for associations with BRD in the additive model were identified on BTA7 (*p* = 1.95 **×** 10^−9^) and BTA4 (*p* = 4.86 **×** 10^−8^). BRD associated SNPs were located within an intron of positional candidate genes early B-cell factor 1 (*EBF1*) on BTA7 and an intron of succinyl-coA:glutarate-CoA transferase (*SUGCT*) on BTA4. *EBF1* was also identified as a positional candidate gene (*p* = 1.31 × 10^−6^) for BRD in the recessive and dominant (*p* = 1.55 **×** 10^−7^) inheritance models. Both positional candidate genes have functions that involve immune response that are consistent with BRD susceptibility. *EBF1*, which was shared between both pre-and post-weaned calves, controls the expression of genes critical for B cell differentiation, signal transduction and inflammatory signaling pathways (30–32). B cells are important to challenge BRD pathogens as they work to reduce inflammation and assist in the production of other lymphocytes and antibodies (33, 34). Reduction in the formation and function of B lymphocytes has been shown to negatively impact the bovine adaptive immune system and increase disease incidence (35). *EBF1* is instrumental in regulating the recombination of V(D) J and maintaining responsiveness of the adaptive immune response against pathogens (36). The *EBF1* protein has demonstrated roles in respiratory disease as it can downregulate the proteasome subunit *β* type 1 (*PSMB1*) protein during porcine reproductive and respiratory syndrome virus (PRRSV) infection to indirectly increase disease susceptibility (37). The downregulation of *PSMB1* reduces the ability of PSMB1 to interact with Nsp12 to inhibit PRRSV infection. Whether *EBF1* would play a similar role in viral infection of BRD pathogens is unknown but further study is warranted. *SUGCT* encodes a protein that is involved in regulating macrophages that are responsible for pro-inflammatory responses to pathogens and mediates the glutarate to glutaryl-CoA reaction in T cells (38, 39). Glutarate is important in a regulator of CD8 + T cell differentiation and increases cytotoxicity against target cells (39). *SUGCT* was also a positional candidate gene in the dominant inheritance model (*p* = 4.95 **×** 10^−8^).

In addition to *SUGCT* and *EBF1*, phosphatidylinositol glycan anchor biosynthesis class Z (*PIGZ*), and nuclear cap binding protein subunit 2 (*NCBP2*) were two additional positional candidate genes with a BRD strongly associated SNP in a 5’ UTR variant of *PIGZ* and an intron of *NCBP2* on BTA1. The *PIGZ* protein is found in the endoplasmic reticulum and adds a fourth mannose to glycosylphophatidylinositol (GPI) during the assembly of GPI anchors. GPI anchors function to attach proteins to the cell surface of many blood cells (40). The *NCBP2* protein forms a heterodimer with NCBP1 to bind to the 5′ cap of pre-mRNA that is needed for pre-mRNA splicing, translation regulation and mRNA decay (41). *NCBP2* is also a positional candidate gene for the dominant inheritance model.

An additional positional candidate gene in the dominant model is cartilage acidic protein 1 (*CRTAC1*) on BTA26. The SNP strongly associated with BRD for this gene was located in an intron. *CRTAC1* codes for a glycoprotein with primary roles in development and repair of the nervous system (42). *CRTAC1* expression in the lung may also be related to neuronal differentiation as *CRTAC1* expression increased in cultured adult and fetal lung epithelial cells treated with isobutyl methylxanthine which induces neuronal differentiation in the lung (43).

In the recessive inheritance model, positional candidate genes that contained a SNP strongly associated with BRD within an exon or intron that has yet to be discussed are located on BTA13 (A*COT8, PLTP*, *CDH22, LOC112449338,* and *WFDC13*), and BTA23 (*PPARD*). In acyl-CoA thioesterase 8 (*ACOT8*), the associated SNP fell within a 3’ UTR variant and in phospholipid transfer protein 1 (*PLTP*), the associated SNP was located within exon 10. The cadherin 22 (*CDH22*) positional candidate gene contained a BRD-associated SNP located in an intron. *ACOT8* and *PLTP* are both involved in lipid metabolism and the immune system (44, 45). *ACOT8* is an important cellular partner of negative factor (Nef) that is thought to be involved in endocytosis and the altering of the cellular environment that influences viral infectivity and replication in HIV infection (44). Early work in bovine immunodeficiency virus (BIV), suggests that there is a lack of Nef compared to human immunodeficiency virus type-1 (HIV-1) (46). Nef functions in a regulatory manner in humans and primates, however in equine, a different protein (S2) functions in place of Nef and similar functions, may be in place within cattle (47, 48). *ACOT8* protein has also been identified as a target for herpes simplex virus 1 (49). The role of *ACOT8* in viral protection against BRD is unknown, but it would be plausible that it may also interact with Nef to affect BRD susceptibility. *PLTP* encodes a lipid transfer protein that is highly expressed in the lungs. It has a role in mucus production and may affect the ability of the lung to remove pathogens (50). *PLTP* expression is increased in chronic obstructive pulmonary disease through cleavage of PLTP by cathepsin G resulting in inflammation of the lung (51, 52). *PLTP* expression is also induced in emphysematous lungs suggesting that a similar role may be possible in the lungs of BRD calves (51). Pulmonary epithelium is comprised of two types of pneumocytes, type 1 and type 2, which function to maintain the airway’s surface. Type 1 pneumocytes comprise alveolar surface area, while type two cells function to secrete a phospholipid surfactant which maintains the surface tension within the lungs (53). Reduced *PLTP* production may decrease surfactant production and pulmonary immune responses and inflammation, which may be observed in cattle with respiratory diseases (54).

The remaining positional candidate genes on BTA13 include *CDH22*, *LOC112449338,* and *WFDC13.* The cadherin 22 (*CDH22*) positional candidate gene contained a BRD-associated SNP located in an intron. *CDH22* is involved in germ line stem cell and tissue formation (55). The cadherin protein family, which includes *CDH22*, produces glycoproteins that function in the cell membrane and aid in cellular adhesion (56). Cadherin proteins assist in producing strong and effective cell-to-cell adhesions that are necessary for the development of all bodily tissues and organs, achieved by forming proteins present in the extracellular matrix that aid in the recognition of other cells (57). For WAP four-disulfide core domain 13 (*WFDC13*), the BRD-associated SNP is located within exon 2. Knockout experiments of *WFDC13* result in infertility due to defects in sperm motility (58). No obvious role for *WFDC13* and BRD is evident. Similarly, the function of *LOC112449338* is unknown as is its possible role in BRD.

Peroxisome proliferator-activated receptor delta (*PPARD*) on BTA23 is the final positional candidate gene strongly associated (*p* < 5 **×** 10^−7^) with BRD in the recessive model. *PPARD* contained BRD-associated SNPs within its introns. *PPARD* serves as a transcriptional repressor, is involved in nuclear receptor signaling and is critical for the establishment of central memory CD8^+^ T cells, which are a key feature of adaptive immunity (59). CD8^+^ memory T cells provide a host with a population of immune cells that are prepared to respond to specific pathogens. CD8^+^ T cells have functions within inflammatory diseases such as asthma and chronic obstructive pulmonary disease, where CD8^+^ T cells have expressed high levels of interleukins and cytokines which influence the onset of inflammation within the lungs (60, 61).

#### 4.2.2 Post-weaned Holstein calves

In post-weaned calves, there were 144 unique positional candidate genes identified. Many of the positional candidate genes that were strongly associated (*p* < 5 **×** 10^−7^) with BRD, contained BRD-associated SNPs within exons or introns, and were shared across inheritance models (Table 2 and Figure 2). There were 23, 17, and 14 positional candidate genes that contained the SNP associated (*p* < 5 **×** 10^−7^) with BRD within either the intron or exon of the gene in the additive, dominant or recessive inheritance models (Supplementary Table 2). The positional candidate genes, among the five loci with the greatest significance for their association with BRD due to an associated SNP in the gene’s exon or intron for each inheritance model, will be further discussed. These positional candidate genes for the additive model are zinc finger and BT domain containing 10 (*ZBTB10*) and zinc finger protein 704 (*ZNF704*) on BTA14, ceramide synthase 6 (*CERS6*) and nitic oxide synthase trafficking (*NOSTRIN*) on BTA2, storkhead box 2 (*STOX2*) on BTA27, and EGF like, fibronectin type II and laminin G domains (*EGFLAM*) on BTA20. The most significant loci in the dominant model included four of the most significant genes in the additive model (*CERS6*, *STOX2*, *NOSTRIN*, and *EGFLAM*), and a single unique gene, ectonucleotide pyrophosphatase/phosphodiesterase 6 (*ENPP6*) on BTA27 (Supplementary Table 2). In the recessive model, two of the eight positional candidate genes from the five most significant loci were shared with those in the additive model (*ZBTB10* and *CERS6*). The recessive model also contained the positional candidate genes EGF domain specific O-linked N-acetylglucosamine transferase (*EOGT*), TAFA chemokine like family member 4 (*FAM19A4*), TATA element modulatory factor 1 (*TMF1*), ubiquitin like modifier activating enzyme 3 (*UBA3*) on BTA22; small integral membrane protein 2 (*SMIM2*) on BTA12; and pappalysin 2 (*PAPPA2*) on BTA16.

*ZBTB10* expresses a transcription factor and is critical for dendritic cell activation and cytokine secretion in mice (62). *ZBTB10* is expressed in the lung and encodes the S1 protein that regulates IL-10, which has anti-inflammatory and immunosuppressive roles (63, 64). *ZBTB10* is also responsible for maintaining genome integrity by binding to hexameric repeats in cells that experience alternative lengthening of telomeres (65). *ZNF704* is also a member of the zinc finger protein family. *ZNF704* is associated with the disruption of circadian rhythm and oncogenesis, though the exact function has not been confirmed (66, 67). Albeituni and Stiban et al. (68) determined that the knockdown of *ZNF704* led to the inhibition of tumor cell growth by increasing the rate of apoptosis in tumor cells and also arrested cell cycle progression in two different cell types.

The second positional candidate gene, *CERS6* which is found in the same locus as *NOSTRIN* on BTA2, was identified as a leading-edge gene enriched for BRD and as a pre-weaned BRD positional candidate gene. Ceramides are essential sphingolipids that work to form the structure of cellular membranes and also assist in cell signaling (69). In humans, *CERS6* ceramides inhibit apoptosis in tumor cells (70). *NOSTRIN*, is responsible for binding to endothelial nitric oxide synthase and assists in the regulation of its movement throughout the body (71). Endothelial nitric oxide synthase functions related to immune system regulation include controlling the onset of vasodilation and inhibiting platelet formation and coagulation (72). Nitric oxide has also been found to reduce the expression of MCP-1, which functions as a chemoattractant, as well as reducing the binding capabilities of leukocytes (72).

*STOX2* is a cofactor with SMAD2/4 and acts to signal TGF-*β*, an essential growth factor among early embryonic stem cells (73). The SNPs associated (*p* = 8.28 × 10^−12^) with BRD is within an intron of *STOX2*. Chen et al. (74) reported that the downregulation of *STOX2* in glioblastoma stem-like cells leads to apoptosis, but the upregulation of *STOX2* in these cells increased the expression of immune suppressing ligands. *EGFLAM* also has functions that have been associated with glioblastoma and cancer cell proliferation (75). Mouse models that knocked down the function of *EGFLAM* showed a reduction in the proliferation, migration and tumor cell invasion in glioblastoma lesions (75). The SNPs associated with BRD are in the intronic regions of *EGFLAM*.

One of the most significant loci associated with BRD in the dominant model in post-weaned heifers contained *ENPP6*. *ENPP6* was the only positional candidate gene that was not shared among the top positional candidate genes for BRD in the dominant and additive models. In mice, *ENPP6* is expressed primarily in brain and liver cells (76). *ENPP6* is a choline-specific phospholipase, which functions to cleave phospholipids essential to the structure and function of cellular membranes (77). One of the phospholipids cleaved by ENPP6 is platelet activating factor, is involved in the immune response to pathogens (76). The functional role of *ENPP6* in response to BRD pathogens has yet to be characterized.

Of the four positional candidate genes located on BTA22 and associated with BRD in the recessive model, the BRD-associated SNPs were located in an intron of *EOGT*, within an intron of *FAM19A4*, within exon 2 of *TMF1*, and within exon nine of *UBA3*. *EOGT* expression is elevated in immune cells that target cancer, but results in immune suppression via reduced numbers of cytotoxic T cells (78). In humans, *EOGT* is downregulated in those who naturally contract respiratory syncytial virus compared to those infected with a research strain (79). Whether *EOGT* would result in immune stimulation in cattle naturally infected with bovine respiratory syncytial virus is unknown, but should be characterized, given the association of this positional candidate gene with BRD in post-weaned heifers. *FAM19A4* promotes the migration and phagocytosis of macrophages (80). *FAM19A4* is upregulated in macrophages and monocytes, aids macrophages in targeting pathogenic cells, and serves as a cytokine (81). *FAM19A4* is also associated with asthma in humans (82). The function of *FAM19A4* in immunity and in the lung, suggests a plausible role for it in cattle experiencing BRD. *TMF1* encodes a golgin protein that functions within the Golgi apparatus and assists in vesicle tethering and transport within the cell (83). Though *TMF1* has not been previously associated with BRD, it has been shown to be associated with a reduction in milk fat in dairy cattle through its regulatory functions on the SREBP1 pathway (84). Both functions of *TMF1* require further validation, however this range of function could pose selection limitations or benefits. The final positional candidate gene of this significant locus associated with BRD on BTA22 is *UBA3. UBA3*, through the process of neddylation, has regulatory roles in adaptive immunity. Neddylation is a process of post-translational protein modification, where the targeted protein is bound to a *NEDD8* protein, and functions similarly to ubiquitination (85). The role of *UBA3* in *UBA3* knockout mice had reduced T cell production identifying that the process of neddylation was importation for T cell survival (86).

*SMIM2* on BTA12 is the next highly associated BRD positional candidate gene for the recessive model in post-weaned heifers. Little is known about the function of *SMIM2*. In humans, *SMIM2* is an RNA coding gene and is predicted to function within membranes (87). A related gene, small integral membrane protein 1 (*SMIM1*), has protein coding functions that assist in regulating the Vel antigen on red blood cells and assists in regulating hemoglobin levels (88). Hemoglobin, and other oxygen-carrying molecules, have functions beyond oxygen transport including inflammatory signaling capabilities and hematopoiesis (89).

*PAPPA2* is the final highly associated BRD positional candidate gene to be discussed for the recessive model, where the associated SNPs are located within an intron on BTA16. *PAPPA2* has multiple functions, including releasing insulin-like growth factor-1 and aids in the regulation of glucose metabolism (90). Mutations within *PAPPA2* have been associated with higher levels of CD4 memory cells and lower levels of Treg cells, suggestive that it limits tumor growth in humans (91). *PAPPA2*’s role in controlling the influx of different immune cells, including CD4 memory cells, may have implications in the immune response raised against BRD pathogens.

### 4.3 Enriched gene sets and leading-edge genes

A single gene set (phospholipid metabolism) was enriched (NES ≥ 3) for BRD in pre-weaned calves, while seven gene sets were enriched (NES ≥ 3) for BRD in the post-weaned calves (Table 3). None of the gene sets between the two calf groups were shared.

Phospholipid metabolism, enriched for BRD in pre-weaned heifers, is a gene set that includes genes encoding lipids that contain phosphoric acid. Phospholipids are instrumental in maintaining the structure of cell membranes and lipids in foods that provide potential health benefits, such as linoleic acid which is involved with reducing inflammation (92). This pathway contains genes with cellular functions that influence respiratory health (including the production of lipid-based pulmonary surfactants), are involved in cellular structure and in immune signaling during pathogen introduction. The production of mucin, which consists mainly of lipids, is necessary to protect pulmonary epithelium and assist in clearing pathogens (93). Phospholipids present in cell membranes also function to send and receive cellular signals and are key in several lung diseases, including idiopathic pulmonary fibrosis (94). It has been demonstrated that free polyunsaturated fatty acids have an antimicrobial effect by acting on bacterial cell membranes by forming metabolites that affect phagocytosis (95). Whether this mechanism is involved in BRD is unknown.

Neuraminidase 2 (*NEU2*) is a positional candidate gene and a leading-edge gene within the pre-weaned BRD population in the phospholipid metabolism gene set. *NEU2* is one of four members of the sialidase family, which removes sialic acid from glycoconjugates (96). Influenza D is a contributor to BRD (97–99), and influenza D utilizes sialic acid receptors (100, 101). Thus, host *NEU2* may function to deplete entry receptors for influenza D virus. Additional work on *NEU2* expression in bovine tissues and its influence on viral entry and replication may clarify its relationship to influenza D virus. In addition, *NEU2* is upregulated in fibrotic lesions in human and mouse lungs but its role in infectious disease in the lung has yet to be characterized (102, 103).

Of the seven gene sets enriched for BRD in post-weaned heifers, three gene sets (G1 phase, cell proliferation, and regulation of cell proliferation) have functions involving cell proliferation that impacts immune, pulmonary, and epithelial cell production (Table 3). The G1 phase gene set contains nine genes (one leading edge gene) that are important in the first phase of the cell cycle, which also is the phase where external signaling has the greatest ability to pause the cellular proliferation process (104). Viruses, such as influenza A in human host cells, have the ability to arrest the cell cycle at G1 in order to preserve conditions for viral replication (105). Similarly, severe acute respiratory syndrome coronavirus and murine coronavirus can also initiate an arrest between G0 and G1 phases to achieve optimal viral replication (105). Although bacteria do not utilize host cell mechanisms for replication, host immune systems do rely on the proliferation of immune cells to combat bacterial and viral infections. The host’s ability to produce phagocytes and leukocytes is essential in clearing pathogen infections such as those seen in BRD (106).

The 120 genes (6 leading edge genes) involved in the cellular proliferation gene set are essential for basic biological functioning and survival (Table 3). Some of these genes are also involved in the regulation of cell proliferation gene set that contains 95 genes (6 leading edge genes). In humans with tissue damage from emphysema, higher rates of cellular apoptosis of affected cells require higher cellular proliferation to replace the damaged tissue (107). A similar scenario was seen among young rats with chronic respiratory disease where pulmonary cell turnover was twice that of healthy rats of the same age (108). Both the regulation of cellular proliferation through the cell cycle and the proliferation of pulmonary cells themselves highlight research opportunities among cattle to examine the impact of cellular proliferation among cattle lungs with and without BRD.

The remaining enriched gene sets (axon guidance mediated by semaphorins, cysteine type peptidase activity, platelet aggregation and signal transduction by L1) for BRD in post-weaned calves have roles in cell signaling. The gene set axon guidance mediated by semaphorins contains 13 genes (2 leading edge genes) that function in neuronal development and axon formation via semaphorin molecules (109). Beyond axonal guidance, semaphorin proteins function in the immune system, where they moderate T-cell activity (110). Semaphorin proteins regulate neutrophil activation, and migration of immune cells in the inflamed lung as asthma has been associated with unregulated semaphorin expression (111, 112). The semaphorin 3A receptor complex interacts with L1 which is a signal transducing receptor (113).

Signal transduction by L1 is a Reactome gene set that consists of 21 genes (3 leading edge genes). This gene set contains a pathway that directly affects neuronal growth and development. Diseases surrounding L1 largely include neurological conditions such as hydrocephaly and Alzheimer’s disease but is also associated with immune disorders such as fetal and neonatal alloimmune thrombocytopenia, platelet-type bleeding disorder, and erythroleukemia, as well as susceptibility to infectious disease such as dermatitis, anthrax, and west Nile virus (114–116).

The Gene Ontology Cytosine type peptidase activity gene set is a pathway that consists of 12 genes (1 leading edge gene) encoding enzymes that hydrolyze peptide bonds in a polypeptide chain (Table 3). Cysteine peptidase genes are involved in adaptive immune responses by regulating T and B lymphocyte apoptosis (117). Cysteine peptidases facilitate antiviral adaptive immune responses during normal and inflammatory conditions in the lung (118). The dysregulation of peptidases is linked to autoimmune diseases as well as bacterial and viral infections (119). Peptidases can be used by viruses, including coronaviruses, to enter host cells and to aid in viral replication, which has made them potential targets for antiviral treatments (120).

The final gene set is from Gene Ontology and is platelet aggregation that contains only two genes (Table 3). Platelet aggregation has been linked to inflammation, and lung diseases (121). The common function of platelets encompasses blood clotting within internal and external wounds. Moreover, their ability to secrete chemokines recruits immune cells and then binds with the immune cells at locations of pathogen infiltration (122). These platelet-immune cell conglomerates can lead to the phagocytosis of pathogens or further inflammation depending on the cells that bind, and coagulation of platelets also inhibits the spread of bacteria throughout the rest of the body (122). In the lung, platelets serve as the first line of defense to combat alveolar damage from viral and bacterial pathogens in acute respiratory distress syndromes (123). Platelets also have been suggested to have a role in the maintenance of preserving alveolar barriers, both through the production of antioxidant enzymes and restricting alveolar permeability to proteins when in a damaged state (123). Platelets have an important role in the immunological functioning of each individual and also assist in the permeability, inflammation, and maintenance of pulmonary epithelium, which could have important implications in cattle with BRD.

Shared leading-edge genes underscore the important role of those genes in susceptibility to BRD in these calves. There is a single leading-edge gene, collagen type IV alpha-3-binding protein (*COL4A3BP*), shared between pre-and post-weaned calves. *COL4A3BP* encodes a ceramide-binding protein, CERT, which is responsible for the transportation of ceramide from its synthesis to its metabolization (124). Ceramides have been linked with cellular membranes and signaling of apoptosis, as they can be converted to ceramide-1-phosphate using ceramide kinase (125). Furthermore, ceramides have been linked to pulmonary inflammation, cystic fibrosis, and emphysema, further highlighting the potential connections between ceramides and BRD (126, 127). A functional connection has been established between ceramides and sphingolipids in the cellular membrane and cystic fibrosis (127). The increase of chronic inflammation within the lungs, influenced via ceramides, increases mucus build-up, inhibits immune responses, and impacts pulmonary structure via cellular apoptosis (127). Pulmonary inflammation and cellular apoptosis are essential elements needed for healthy and properly functioning respiratory systems, and an improper regulation of these mechanisms could lead to an increased risk of contracting diseases such as cystic fibrosis.

In post-weaned calves, there is one leading-edge gene, p21-activated kinase 1 (*PAK1*) gene on BTA11, that is shared in four of the seven enriched gene sets (NES ≥ 3.0; Table 3). The PAK family of proteins are comprised of six protein members (128). PAK proteins (1–3) play a role in cytoskeleton structure, cell proliferation, and preventing apoptosis (129, 130). In humans, *PAK1* proteins have been linked with increased inflammation within the lungs when infected with the COVID-19 virus (131). Whether *PAK1* expression also leads to lung inflammation in cattle when exposed to the BRD pathogen, bovine coronavirus, has yet to be established.

Five leading-edge genes, cyclin-dependent kinase inhibitor 2 B (*CDKN2B*), cathepsin H (*CTSH*), casein kinase 2 alpha 1 (*CSNK2A1*), proto-oncogene tyrosine-protein kinase Fyn (*FYN*), and integrin subunit beta 1 (*ITGB1*), were shared in three of the seven gene sets enriched (NES ≥ 3.0) for BRD. *CDKN2B* encodes a protein that inhibits cell cycle progression and has been associated with idiopathic pulmonary fibrosis and amplifies sepsis-induced lung injury (132–134). *CTSH* has a strong expression in type 2 pneumocytes, and the production of pulmonary surfactant (135). *CTSH* is differentially expressed in the lungs of individuals at risk for lung adenocarcinoma when macrophages were examined suggesting a role in cellular proliferation in the lung (136). *CTSH* was also identified as a differentially expressed gene for bovine respiratory disease in a population of Xinjiang calves (137). *CSNK2A1* is one of two genes encoding CK2 protein kinases, which function to phosphorylate proteins and can be exploited by viruses who utilize phosphorylated proteins to support viral proliferation (138). *CSNK2A1* phosphorylates acid proteins including many transcription factors such as NF-kappa-B*, STAT1, CREB1, IRF1, IRF2, ATF1, ATF4, SRF, MAX, JUN, FOS, MYC* and *MYB* as well as proteins involved in immune responses to viral life cycles of Epstein–Barr virus, herpes simplex virus, hepatitis B virus, chronic hepatitis C virus, human immunodeficiency virus, cytomegalovirus and human papillomavirus (139–143). *FYN* is expressed in T cells and has key roles in the development, selection and maintenance of naïve and peripheral T cells (144, 145). *FYN* has suggested roles in mice with negative regulation of pulmonary inflammation due to its influence on T-cell signaling (146). Lastly, *ITGB1* has been expressed within macrophages and types 1 and 2 pneumocytes, both having implications with pulmonary immunity and maintaining alveolar interface (147). These genes share roles in immune response highlighting opportunities for selection in BRD.

There was also a single gene, SUMO specific peptidase 7 (*SENP7*), that was identified as a positional candidate and leading-edge genes for BRD in post-weaned calves. The post-translational removal of small ubiquitin-like modifiers (SUMOs) is regulated by the *SENP* family of genes (148, 149). *SENP7* has a role in the formation of heterochromatin during mitosis. Reduced expression of *SENP7* can lead to alterations in chromatin structure, as *SENP7* is needed for chromatin availability for DNA damage repair (150–152). Genes involved in the regulation of SUMOs have roles in immune cell activation and identifying the presence of, and mounting of, defenses against pathogens (153). Guo et al. (154) found a connection with *SENP7* and arthrogryposis multiplex congenita, where one of the symptoms of the fatal disease is early respiratory failure.

Two genes, *CERS6* and protein kinase CGMP-dependent 1 (*PRKG1*), were shared as enriched/associated genes with BRD within both the pre-and post-weaned calves. *CERS6*, discussed previously due to being highly associated within the post-weaned BRD population, assists in regulating cellular structure and signaling. *PRKG1* is located on BTA26 and was a leading-edge gene in pre-weaned heifers and a positional candidate gene in post-weaned heifers. The kinase produced by *PRKG1* is responsible for managing smooth muscle relaxation (155). In humans, *PRKG1* has functions in bronchodilation and asthma (156–158). Both genes and the role of ceramides and smooth muscle function, provide opportunities for selection for enhanced BRD resistance in cattle.

There was also a shared leading-edge gene, vitamin D receptor (*VDR*), that was identified within the post-weaned BRD population. *VDR* was a leading-edge gene within two enriched gene sets (cell proliferation and the regulation of cell proliferation) within the post-weaned BRD population in this study and was also a leading-edge gene among the enriched steroid binding gene set identified by Kiser et al. (27). *VDR* is responsible for binding vitamin D, which is known to have multiple biological roles including assisting in the regulation of cell cycle control as well as having abilities to repress the expression of T-cells and cytokine producing genes (159, 160). These shared genes highlight the opportunity to identify potential regions for selection for BRD resistance across breed, region, or farm operation.

## 5 Conclusion

This study identified 50 loci strongly associated (*p* < 5 **×** 10^−7^) with BRD that contained 65 unique positional candidate genes, and one gene set and 86 leading-edge genes enriched (NES ≥ 3.0) for BRD in pre-weaned calves. In post-weaned calves, 106 loci and 144 positional candidate genes were strongly associated (*p* < 5 **×** 10^−7^) with BRD and seven gene sets and 162 unique leading-edge genes were enriched (NES ≥ 3.0) for BRD. There was also a single positional candidate gene (*CTSH*) and a single leading-edge gene (*VDR*) that were shared with previous BRD work. The genes identified in these analyses highlight genetic regulatory processes of the immune system, cell growth and proliferation, and cellular communication. The loci and genes associated with this population provide further insight into the genomic susceptibility of BRD in dairy cattle and offer potential targets for genomic selection to reduce the morbidity and mortality of this common disease.

## Data Availability

The datasets used within this study have been published to online repositories. The repository and data information can be found via the following link: https://doi.org/10.17605/OSF.IO/QYM4E.

## Footnotes

^1^ https://bovinegenome.org

^2^ https://www.animalgenome.org/repository/cattle/UMC_bovine_coordinates/

^3^ http://www.genecarta.com/

^4^ http://www.geneontology.com

^5^ http://www.genome.jp.kegg

^6^ http://www.pantherdb.org

^7^ http://www.reactome.org
