# Supplementary material for: Genomic regions associated with bovine respiratory disease in pacific northwest Holstein cattle
Source: Front Vet Sci. 2025 Jul 31;12:1637087. doi: 10.3389/fvets.2025.1637087 (PMC12350131; doi:10.3389/fvets.2025.1637087)
Supplement: Supplementary file 1 [file Table_1.docx]

**Supplemental Table 1**. Genome wide association analysis of pre-weaned Holstein heifer calves listing loci associated (*P* < 1 x 10^-5^) with bovine respiratory disease.

| **BTA^1^** | **# Associated SNPs^2^** | **Mb^3^** | **P-Value^4^** | **Proportion of Variance Explained^5^** | **Inheritance Model^6^** | **Positional Candidate Genes^7^** |
| --- | --- | --- | --- | --- | --- | --- |
| 1 | 1 | 38 | 2.71 x 10^-6^ | 0.53% | Additive | - |
| 1 | 3 | 71 | 4.56 x 10^-6^ | 0.50% | Additive | ***NCBP2****, NCBP2-AS2,* ***PIGZ,*** *SENP5* |
| 1 | 1 | 138 | 8.14 x 10^-6^ | 0.48% | Additive | ***CPNE4****, TRNAS-GCU* |
| 2 | 1 | 102 | 8.46 x 10^-6^ | 0.47% | Additive | *SPAG16,* ***VWC2L*** |
| 3 | 1 | 77 | 7.67 x 10^-7^ | 0.58% | Additive | *-* |
| 4 | 3 | 80 | **4.86** **x 10^-8^** | 0.71% | Additive | ***SUGCT*** |
| 4 | 1 | 87 | 2.72 x 10^-6^ | 0.53% | Additive | *CADPS2, FEZF1, LOC112446415* |
| 5 | 8 | 46 | **2.37** **x 10^-7^** | 0.64% | Additive | *DYRK2, LOC112446699* |
| 7 | 1 | 71 | **1.95** **x 10^-9^** | 0.86% | Additive | ***EBF1*** |
| 9 | 8 | 41 | 3.03 x 10^-6^ | 0.52% | Additive | ***CCDC162P****, CEP57L1, LOC112448040,* ***LOC112448041*** |
| 9 | 3 | 55 | **4.46** **x 10^-7^** | 0.61% | Additive | *-* |
| 9 | 1 | 92 | 5.64 x 10^-6^ | 0.49% | Additive | ***TFB1M*** |
| 10 | 1 | 84 | 8.57 x 10^-6^ | 0.47% | Additive | *DCAF4, DPF3* |
| 11 | 6 | 66 | 8.43 x 10^-6^ | 0.52% | Additive | *-* |
| 17 | 1 | 14 | 8.84 x 10^-6^ | 0.47% | Additive | *-* |
| 21 | 1 | 62 | 8.72 x 10^-6^ | 0.47% | Additive | *-* |
| 24 | 2 | 8 | 6.35 x 10^-6^ | 0.56% | Additive | ***DOK6*** |
| 26 | 3 | 7 | 9.22 x 10^-6^ | 0.48% | Additive | ***PRKG1*** |
| 26 | 3 | 19 | 5.62 x 10^-6^ | 0.49% | Additive | *-* |
| X | 2 | 2-51 | 4.89 x 10^-6^ | 0.56% | Additive | *-* |
| 1 | 3 | 71 | **2.25** **x 10^-7^** | 0.65% | Dominant | ***NCBP2****, NCBP2-AS2,* ***PIGZ****, SENP5* |
| 3 | 1 | 77 | 6.27 x 10^-6^ | 0.49% | Dominant | *-* |
| 3 | 4 | 113 | 6.52 x 10^-6^ | 0.49% | Dominant | *INPP5D,* ***NEU2****, NGEF* |
| 4 | 3 | 80 | **4.95** **x 10^-8^** | 0.71% | Dominant | ***SUGCT*** |
| 5 | 7 | 46 | 7.58 x 10^-7^ | 0.59% | Dominant | *DYRK2, LOC112446699* |
| 7 | 1 | 71 | **1.55** **x 10^-7^** | 0.66% | Dominant | ***EBF1*** |
| 7 | 2 | 78 | 8.47 x 10^-6^ | 0.51% | Dominant | *-* |
| 8 | 3 | 107 | 7.74 x 10^-6^ | 0.50% | Dominant | *-* |
| 9 | 9 | 41 | 8.17 x 10^-6^ | 0.57% | Dominant | *CEP57L1,* ***CCDC162P****, LOC112448040,* ***LOC112448041*** |
| 9 | 4 | 55-64 | **4.67** **x 10^-7^** | 0.61% | Dominant | *-* |
| 9 | 1 | 92 | 6.49 x 10^-6^ | 0.51% | Dominant | ***TFB1M*** |
| 11 | 2 | 66 | 4.51 x 10^-6^ | 0.51% | Dominant | *-* |
| 15 | 2 | 72 | 7.19 x 10^-7^ | 0.59% | Dominant | *-* |
| 24 | 1 | 8 | 3.30 x 10^-6^ | 0.52% | Dominant | ***DOK6*** |
| 26 | 1 | 7 | 5.27 x 10^-6^ | 0.50% | Dominant | ***PRKG1*** |
| 26 | 3 | 19 | **1.03** **x 10^-7^** | 0.68% | Dominant | ***CRTAC1*** |
| X | 1 | 2 | **1.99** **x 10^-7^** | 0.65% | Dominant | *-* |
| 2 | 1 | 24 | 6.38 x 10^-6^ | 0.49% | Recessive | ***RAPGEF4*** |
| 5 | 3 | 54 | 9.63 x 10^-6^ | 0.47% | Recessive | *-* |
| 6 | 1 | 116 | 2.34 x 10^-6^ | 0.53% | Recessive | *LOC104968946,* ***ZFYVE28*** |
| 7 | 1 | 4 | 3.48 x 10^-6^ | 0.52% | Recessive | *COMP,* ***CRTC1****, LOC112447583* |
| 7 | 2 | 71-74 | 8.28 x 10^-6^ | 0.56% | Recessive | ***EBF1*** |
| 8 | 2 | 3 | 2.06 x 10^-6^ | 0.54% | Recessive | ***LOC104969247*** |
| 13 | 6 | 75 | **2.73** **x 10^-9^** | 0.85% | Recessive | ***ACOT8, CDH22,*** *CTSA, DNTTIP1, LOC104973895,* ***LOC112449338,*** *LOC112449416, NEURL2, PCIF1,* ***PLTP****, SNX21, SPATA25, TNNC2, UBE2C, WFDC10A, WFDC11,* ***WFDC13****, WFDC9, ZSWIM1, ZSWIM3* |
| 14 | 2 | 27 | **3.36** **x 10^-7^** | 0.62% | Recessive | *-* |
| 18 | 1 | 2 | 2.63 x 10^-6^ | 0.53% | Recessive | *COG4, FUK, ST3GAL2* |
| 18 | 4 | 8 | 9.03 x 10^-6^ | 0.47% | Recessive | ***LOC112442253, PKD1L2*** |
| 23 | 5 | 9 | **4.41** **x 10^-7^** | 0.64% | Recessive | ***PPARD*** |
| 26 | 9 | 7 | 7.81 x 10^-6^ | 0.50% | Recessive | ***PRKG1*** |
| 28 | 3 | 6 | 7.80 x 10^-6^ | 0.48% | Recessive | ***PCNX2*** |

^1^*Bos taurus* chromosome (BTA) number of where the locus is associated with pre-weaned BRD. ^2^Number of single nucleotide polymorphisms (SNPs) present within each associated locus. ^3^Megabase (Mb) position of each locus. ^4^The uncorrected p-value for the lead SNP at each locus. Bolded values were strongly associated with pre-weaned BRD. ^5^The proportion of variance explained (%) is listed for the most significant SNP within each associated locus. ^6^The inheritance model where each locus was associated with pre-weaned BRD. ^7^Positional candidate genes are genes that were identified within ± 30 kb (5’ and 3’) of associated SNPs. Bolded positional candidate genes have one of the associated SNPs located within it.

**Supplemental Table 2**. Genome wide association analysis of post-weaned Holstein heifer calves listing loci associated (*P* < 1 x 10^-5^) with bovine respiratory disease.

| **BTA^1^** | **# Associated SNPs^2^** | **Mb^3^** | **P-Value^4^** | **Proportion of Variance Explained^5^** | **Inheritance Model^6^** | **Positional Candidate Genes^7^** |
| --- | --- | --- | --- | --- | --- | --- |
| 1 | 5 | 19 | **8.88** **x 10^-8^** | 0.55% | Additive | *CHODL* |
| 1 | 1 | 46 | **5.16** **x 10^-9^** | 0.65% | Additive | ***SENP7*** |
| 1 | 1 | 93 | 5.54 x 10^-7^ | 0.48% | Additive | *NAALADL2* |
| 2 | 43 | 27 | **5.67** **x 10^-13^** | 0.99% | Additive | ***CERS6****, LOC112443601,* ***NOSTRIN****, SPC25* |
| 2 | 2 | 56 | **3.20** **x 10^-07^** | 0.50% | Additive | *TRNAC-GCA* |
| 2 | 1 | 68 | **1.50** **x 10^-07^** | 0.53% | Additive | *LOC112443654* |
| 2 | 1 | 102 | 5.97 x 10^-6^ | 0.39% | Additive | ***SPAG16*** |
| 3 | 37 | 19 | 8.18 x 10^-6^ | 0.44% | Additive | ***CGN****, LOC100297233,* ***LOC112445949****, LOC112446124,* ***LOC787530****,* ***SNX27****,* ***TUFT1*** |
| 5 | 2 | 83 | **4.57** **x 10^-7^** | 0.49% | Additive | ***ITPR2*** |
| 7 | 3 | 41 | 2.61 x 10^-6^ | 0.42% | Additive | *LOC518816, LOC526765, LOC787758, OR2W3, TRIM58* |
| 8 | 2 | 24 | **1.82** **x 10^-8^** | 0.61% | Additive | ***FOCAD****, MIR491* |
| 8 | 8 | 33 | **3.15** **x 10^-7^** | 0.50% | Additive | *LOC112447904* |
| 8 | 1 | 105 | 6.05 x 10^-7^ | 0.48% | Additive | *-* |
| 9 | 5 | 33 | **3.66** x 10**^-11^** | 0.84% | Additive | ***DCBLD1****, LOC112448032* |
| 9 | 1 | 57 | **2.51** **x 10^-7^** | 0.51% | Additive | *-* |
| 10 | 2 | 22 | 6.10 x 10^-6^ | 0.39% | Additive | ***LOC100336282****, LOC107132830, LOC112448409, LOC112448411* |
| 11 | 1 | 42 | 4.15 x 10^-6^ | 0.41% | Additive | *-* |
| 11 | 7 | 47 | **3.91** **x 10^-8^** | 0.58% | Additive | ***LOC100294952*** |
| 11 | 1 | 47 | 2.08 x 10^-6^ | 0.43% | Additive | ***EIF2AK3****, TEX37* |
| 11 | 1 | 47 | 1.62 x 10^-6^ | 0.44% | Additive | *LOC783227* |
| 11 | 1 | 66 | 2.60 x 10^-6^ | 0.42% | Additive | *-* |
| 12 | 24 | 14 | **2.49** **x 10^-10^** | 0.77% | Additive | *LACC1, LOC112449035,* ***SERP2****,* ***SMIM2****, TSC22D1* |
| 12 | 2 | 42 | 5.67 x 10^-6^ | 0.39% | Additive | *-* |
| 13 | 1 | 5 | **9.83** **x 10^-8^** | 0.54% | Additive | *-* |
| 13 | 1 | 10 | **1.80** **x 10^-11^** | 0.86% | Additive | *-* |
| 13 | 3 | 70 | 5.71 x 10^-7^ | 0.48% | Additive | ***CHD6****, LOC112449381* |
| 14 | 6 | 44 | **6.95** **x 10^-14^** | 1.07% | Additive | *LOC100295528, LOC112449520, LOC785035,* ***ZBTB10****,* ***ZNF704*** |
| 14 | 1 | 77 | 6.85 x 10^-6^ | 0.39% | Additive | *-* |
| 15 | 2 | 50 | 9.48 x 10^-7^ | 0.46% | Additive | *LOC112441490, LOC618010,* ***LOC784976****, LOC785036, OR51E2* |
| 16 | 3 | 1 | 6.64 x 10^-6^ | 0.39% | Additive | ***FMOD****, LOC107133214* |
| 18 | 4 | 21 | 1.37 x 10^-6^ | 0.45% | Additive | ***CHD9****, LOC112442284,* ***TOX3****, TRNAC-ACA* |
| 18 | 3 | 51 | **4.38** **x 10^-8^** | 0.57% | Additive | ***CNFN****, LIPE,* ***MEGF8*** |
| 18 | 1 | 61 | 2.92 x 10^-6^ | 0.42% | Additive | *LOC504704,* ***LOC506868****, LOC615600, NLRP12* |
| 20 | 3 | 36 | **8.26** **x 10^-10^** | 0.72% | Additive | ***EGFLAM****, LOC101905359* |
| 22 | 12 | 33 | **4.66** **x 10^-7^** | 0.49% | Additive | *ARL6IP5,* ***EOGT****,* ***FAM19A4****,* ***TMF1****,* ***UBA3*** |
| 22 | 4 | 41 | 5.87 x 10^-7^ | 0.48% | Additive | *-* |
| 23 | 8 | 28 | 8.60 x 10^-7^ | 0.46% | Additive | *ABHD16A,* ***C23H6orf10****,* ***C23H6orf15****, CCHCR1, CDSN, CLIC1, DDAH2, DDR1, LOC100141101, LOC112443865, LOC112443912, LOC616942, LY6G5B, LY6G5C,* ***LY6G6E****,* ***LY6G6F****, MPIG6B, MSH5, PSORS1C2* |
| 26 | 1 | 29 | **2.63** **x 10^-7^** | 0.51 | Additive | *-* |
| 27 | 3 | 14 | **7.43** **x 10^-8^** | 0.57% | Additive | *DCTD,* ***LOC100848319*** |
| 27 | 2 | 14 | **4.77** **x 10^-7^** | 0.49% | Additive | *LOC112444630, LOC536739,* ***WWC2*** |
| 27 | 12 | 14 | **9.64** **x 10^-12^** | 0.89% | Additive | *CASP3, CDKN2AIP,* ***ENPP6,*** *ING2, LOC101903828, LOC101904332, LOC101907089, LOC104976048,* ***PRIMPOL, STOX2,*** *TRAPPC11* |
| 29 | 1 | 10 | 1.51 x 10^-6^ | 0.44% | Additive | ***CCDC83*** |
| 1 | 5 | 19 | **2.63** **x 10^-7^** | 0.51% | Dominant | *CHODL* |
| 1 | 2 | 45 | **1.50** **x 10^-8^** | 0.61% | Dominant | *ABI3BP,* ***SENP7*** |
| 1 | 1 | 93 | 8.44 x 10^-6^ | 0.38% | Dominant | *NAALADL2* |
| 2 | 42 | 27 | **1.52** **x 10^-14^** | 1.13% | Dominant | ***CERS6****, LOC112443601,* ***NOSTRIN****, SPC25* |
| 2 | 1 | 95 | 9.25 x 10^-6^ | 0.38% | Dominant | ***DYTN*** |
| 3 | 21 | 19 | 6.14 x 10^-6^ | 0.39% | Dominant | *CGN, LOC100297233,* ***LOC112445949****, LOC112446124,* ***LOC787530****,* ***SNX27****,* ***TUFT1*** |
| 7 | 1 | 71 | 6.26 x 10^-6^ | 0.39% | Dominant | ***EBF1*** |
| 8 | 10 | 23-32 | **3.04** **x 10^-8^** | 0.61% | Dominant | *CDKN2A, CDKN2B,* ***FOCAD****, LOC101907577, LOC112447904, MIR491* |
| 9 | 5 | 33 | **7.11** **x 10^-10^** | 0.73% | Dominant | ***DCBLD1****, LOC112448032* |
| 9 | 1 | 57 | **2.41** **x 10^-8^** | 0.60% | Dominant | *-* |
| 10 | 2 | 22 | 1.49 x 10^-6^ | 0.44% | Dominant | ***LOC100336282****, LOC107132830, LOC112448409, LOC112448411* |
| 11 | 1 | 42 | 1.93 x 10^-6^ | 0.43% | Dominant | *-* |
| 11 | 7 | 47 | **3.79** **x 10^-8^** | 0.58% | Dominant | *LOC100294952, LOC784634, RPIA* |
| 11 | 2 | 66 | 5.39 x 10^-7^ | 0.48% | Dominant | *-* |
| 12 | 14 | 14 | **7.22** **x 10^-10^** | 0.78% | Dominant | *LOC112449035,* ***SERP2****, TSC22D1* |
| 13 | 1 | 5 | 6.16 x 10^-6^ | 0.39% | Dominant | *-* |
| 13 | 1 | 5 | **1.26** **x 10^-8^** | 0.62% | Dominant | *-* |
| 13 | 8 | 10 | **9.96** **x 10^-12^** | 0.89% | Dominant | *-* |
| 13 | 5 | 70 | **7.67** **x 10^-8^** | 0.61% | Dominant | ***CHD6****, LOC112449381* |
| 14 | 5 | 44 | **2.39** **x 10^-7^** | 0.51% | Dominant | *LOC112449520, LOC785035,* ***ZBTB10****,* ***ZNF704*** |
| 14 | 1 | 77 | 6.07 x 10^-7^ | 0.48% | Dominant | *-* |
| 15 | 3 | 50 | **4.23** **x 10^-7^** | 0.49% | Dominant | *LOC112441490, LOC112441705, LOC618010, LOC782428,* ***LOC784976****, LOC785036,* ***LOC788946****, OR51A7, OR51E2, OR51T1* |
| 15 | 2 | 72 | 7.61 x 10^-6^ | 0.38% | Dominant | *-* |
| 16 | 2 | 71 | 4.91 x 10^-6^ | 0.40% | Dominant | *-* |
| 17 | 1 | 63 | 7.23 x 10^-6^ | 0.39% | Dominant | *ACADS, TRNAG-UCC, UNC119B* |
| 18 | 1 | 21 | 7.34 x 10^-6^ | 0.39% | Dominant | ***TOX3*** |
| 20 | 2 | 36 | **8.23** **x 10^-9^** | 0.64% | Dominant | ***EGFLAM*** |
| 20 | 1 | 56 | 5.29 x 10^-6^ | 0.40% | Dominant | ***LOC101905359*** |
| 21 | 1 | 31 | 1.34 x 10^-6^ | 0.45% | Dominant | ***HYKK****, IREB2, LOC112443142, PSMA4* |
| 22 | 1 | 33 | 1.83 x 10^-6^ | 0.44% | Dominant | ***EOGT****, TMF1* |
| 22 | 1 | 33 | **1.39** **x 10^-7^** | 0.53% | Dominant | ***FAM19A4*** |
| 22 | 1 | 41 | 8.64 x 10^-6^ | 0.39% | Dominant | ***FHIT*** |
| 22 | 1 | 41 | 2.87 x 10^-6^ | 0.42% | Dominant | ***FHIT*** |
| 23 | 1 | 27 | 4.56 x 10^-6^ | 0.40% | Dominant | ***C23H6orf10*** |
| 23 | 1 | 28 | 1.83 x 10^-6^ | 0.44% | Dominant | *ABHD16A, CLIC1, DDAH2, LY6G5B, LY6G5C, LY6G6C,* ***LY6G6E****,* ***LY6G6F****, MPIG6B, MSH5* |
| 26 | 7 | 29 | **9.12** **x 10^-9^** | 0.63% | Dominant | *-* |
| 27 | 17 | 14 | **2.12** **x 10^-10^** | 0.78% | Dominant | *DCTD,* ***ENPP6****,* ***LOC100848319****, LOC101904332, LOC104976048, LOC112444630, LOC536739,* ***STOX2****, TRAPPC11,* ***WWC2*** |
| 0 | 1 | 0 | **1.58** **x 10^-8^** | 0.61% | Recessive | *-* |
| 2 | 1 | 27 | **1.83** **x 10^-10^** | 0.78% | Recessive | ***CERS6*** |
| 2 | 1 | 68 | 4.49 x 10^-6^ | 0.40% | Recessive | *LOC112443654* |
| 3 | 2 | 119 | 5.06 x 10^-6^ | 0.40% | Recessive | *-* |
| 4 | 1 | 36 | 7.35 x 10^-7^ | 0.47% | Recessive | *SEMA3A* |
| 5 | 3 | 1 | **2.09** **x 10^-7^** | 0.52% | Recessive | *-* |
| 5 | 2 | 83 | **1.51** **x 10^-7^** | 0.53% | Recessive | ***ITPR2*** |
| 7 | 6 | 103 | 8.91 x 10^-6^ | 0.38% | Recessive | *LOC112447499, LOC112447532, LOC112447595* |
| 8 | 1 | 33 | **1.09** **x 10^-10^** | 0.80% | Recessive | *-* |
| 8 | 1 | 105 | 6.04 x 10^-6^ | 0.39% | Recessive | *-* |
| 9 | 1 | 24 | 2.48 x 10^-6^ | 0.42% | Recessive | ***SNAP91*** |
| 10 | 2 | 102 | 1.95 x 10^-6^ | 0.43% | Recessive | ***EFCAB11*** |
| 12 | 1 | 14 | **2.26** **x 10^-13^** | 1.02% | Recessive | *-* |
| 12 | 1 | 14 | **4.86** **x 10^-10^** | 0.74% | Recessive | ***SMIM2*** |
| 12 | 1 | 15 | 6.05 x 10^-6^ | 0.39% | Recessive | *LOC112449035* |
| 13 | 2 | 70 | **8.22** **x 10^-8^** | 0.55% | Recessive | ***CHD6*** |
| 14 | 3 | 44 | **2.82** **x 10^-17^** | 1.36% | Recessive | *LOC100295528,* *LOC101905394,* ***ZBTB10****,* ***ZNF704*** |
| 16 | 2 | 58 | **1.08** **x 10^-9^** | 0.71% | Recessive | *LOC112441790,* ***PAPPA2*** |
| 18 | 1 | 21 | 8.05 x 10^-6^ | 0.38% | Recessive | *-* |
| 22 | 24 | 33 | **1.68** **x 10^-15^** | 1.21% | Recessive | *ARL6IP5,* ***EOGT****,* ***FAM19A4****, FRMD4B, LOC112443429,* ***TMF1****,* ***UBA3*** |
| 23 | 1 | 6 | **2.01** **x 10^-7^** | 0.52% | Recessive | *-* |
| 27 | 1 | 13 | **1.57** **x 10^-7^** | 0.53% | Recessive | ***TENM3*** |
| 27 | 1 | 14 | 5.65 x 10^-6^ | 0.39% | Recessive | ***STOX2*** |
| 27 | 3 | 14 | 3.11 x 10^-6^ | 0.42% | Recessive | *ENPP6,* ***STOX2*** |
| 28 | 1 | 8 | 7.07 x 10^-6^ | 0.39% | Recessive | *-* |
| 29 | 1 | 10 | 1.91 x 10^-6^ | 0.43% | Recessive | ***CCDC83*** |

^1^*Bos taurus* chromosome (BTA) number of where the locus is associated with post-weaned BRD. ^2^Number of single nucleotide polymorphisms (SNPs) present within each associated locus. ^3^Megabase (Mb) position of each locus. ^4^The uncorrected p-value for the lead SNP for each locus. Bolded values were strongly associated (p < 5 x 10^-7^) with post-weaned BRD ^5^The proportion of variance explained (%) is listed for the most significant SNP within each associated locus. ^6^The inheritance model where each locus was associated with pre-weaned BRD. ^7^Positional candidate genes are genes that were identified within ± 30 kb (5’ and 3’) of associated SNPs. Bolded positional candidate genes have one of the associated SNPs located within it.
